# Thyroid cancer and its associations with dietary quality in a 1:1 matched case–control study

**DOI:** 10.1017/S0007114522000836

**Published:** 2023-01-28

**Authors:** Manman Xia, Jiajie Zang, Zhengyuan Wang, Jiadong Wang, Yi Wu, Meixia Liu, Zehuan Shi, Qi Song, Xueying Cui, Xiaodong Jia, Fan Wu

**Affiliations:** 1 Division of Infectious Disease Prevention and Control, Songjiang District for Disease Control and Prevention, Shanghai, People’s Republic of China; 2 Division of Health Risk Factors Monitoring and Control, Shanghai Municipal Center for Disease Control and Prevention, Shanghai, People’s Republic of China; 3 Department of Head and Neck Surgery, Renji Hospital, School of Medicine, Shanghai Jiao Tong University, Shanghai, People’s Republic of China; 4 Shanghai Tumor Hospital, Shanghai, People’s Republic of China; 5 Shanghai Institutes of Preventive Medicine, Shanghai, People’s Republic of China; 6 Shanghai Medical college, Fudan University, Shanghai 200032, People’s Republic of China

**Keywords:** Thyroid cancer, The Chinese Health Dietary Index, Diet-quality score, Case-control study

## Abstract

Thyroid cancer (TC) incidence has increased greatly during the past decades with a few established risk factors, while no study is available that has assessed the association of the Chinese Health Dietary Index (CHDI) with TC. We conducted a 1:1 matched case–control study in two hospitals in Shanghai, China. Diet-quality scores were calculated according to CHDI using a validated and reliable FFQ. Conditional logistic regression analysis and restricted cubic spline analysis were used to reveal potential associations between CHDI score and TC risk. A total of 414 pairs of historically confirmed TC patients and healthy controls were recruited from November 2012 to December 2015. The total score of cases and controls were 67·5 and 72·8, respectively (*P* < 0·001). The median score of total vegetables, fruit, diary products, dark green and orange vegetables, fish, shellfish and mollusk, soyabean, whole grains, dry bean and tuber in cases was significantly lower than those in controls. Compared with the reference group (≤60 points), the average (60–80 points) and high (≥80 points) levels of the CHDI score were associated with a reduced risk of TC (OR: 0·40, 95 % CI 0·26, 0·63 for 60–80 points; OR: 0·22, 95 % CI 0·12, 0·38 for ≥80 points). In age-stratiﬁed analyses, the favourable association remained signiﬁcant among participants who are younger than 50 years old. Our data suggested that high diet quality as determined by CHDI was associated with lower risk of TC.

Thyroid cancer (TC) is the most common cancer of the endocrine system, accounting for 1–2 % of all new cancers diagnosed each year worldwide^(1,2)^. With TC being the fifth most common cancer diagnosed in women in the USA in 2017 and the seventh most common cancer diagnosed in Australian women in 2017^(3,4)^, a worldwide increase in the incidence of TC has occurred. However, much higher incidences have been observed in southern China, especially in Shanghai (incidence, 36·06/100 000) in 2014^(5)^. In Shanghai, the incidence of TC became the first cancer among women, with approximately 77·2 cases per 100 000 women in 2018^(6)^, the burden of the diagnosis and treatment on individuals as well as the health care system is significant. More efforts are needed to explore the risk factors of TC to allow more effective prevention in the Shanghai population.

Several risk factors have been associated with TC, including non-modifiable factors such as genetics, ionising radiation in childhood, age and sex, and modifiable factors including the food and the diet^(7,8)^. Previous epidemiology studies have shown an association between the risk of TC and the consumption of seafood^(9)^, preserved meat^(10,11)^, cruciferous vegetables (cabbage, broccoli, cauliflower, etc.), starchy food^(12)^ and fresh fruit and vegetables^(13)^. While it has long been suspected that TC has a nutritional aetiology, the relationship between dietary components and TC remains unclear^(14)^. It has been proposed that examination of overall diet food may give a more accurate view of the association between diet and cancer and provide insight into the complex interactions between different foods^(15)^. Currently, accumulating studies have developed overall dietary scores to comprehensively evaluate the role of diet in the risks of types of cancer, for example, laryngeal cancer^(16)^, pancreatic cancer^(17)^, prostate cancer^(18)^, oesophageal cancer^(19)^ and colorectal cancer^(20)^, but its role in TC has not been fully investigated.

To date, limited literature has investigated the association between dietary patterns and the risk of TC with inconsistent findings^(21–24)^. A case–control study from Hangzhou, China found a protective effect of compliance with the seafood and cereal pattern on TC^(21)^. Similarly, in the population-based case–control study, a dietary pattern high in fruit and vegetables was reported to be inversely correlated with risks of both overall TC and papillary TC^(22)^. Conversely, a study from Middle Eastern country observed that the Western dietary pattern including high intake of meat and fried or roasted meat, fish, high fat dairy products, olivier salad, potato, snacks and nuts, and sugar was associated with increased risk of differentiated TC^(23)^. A hospital-based case–control study from Catania highlighted an increased risk of TC associated with the consumption of some food items (starchy foods/refined cereal, products rich in salt and fat, and sweets) and caffeinated drinks^(24)^. The Chinese Health Dietary Index (CHDI), based on the 2016 Chinese Dietary Guidelines and Dietary Pyramid, is the latest comprehensive approach to assess the quantity and quality of diet in Chinese population and has proved useful in surveilling national nutrition transitions and epidemiological trends^(25)^. However, to our knowledge, no study has yet reported associations between overall diet-quality scores and the risk of TC.

Therefore, we performed a 1:1 matched and hospital-based case–control study to assess the association between CHDI and the risk of TC in a high-incidence population in the coastal area of China, and whether the association differs depending on sex and age.

## Materials and methods

### Study population

This matched hospital-based case–control study was conducted between November 2012 and December 2015 in Shanghai, China. TC patients were selected by using convenience-sampling method from two hospitals (Renji Hospital of Shanghai Jiaotong University School of Medicine and Fudan University Shanghai Cancer Center) from November 2012 to December 2015. The diagnosis of each new case was histologically confirmed in two hospitals (International Classification of Disease for Oncology, Third Edition diagnostic codes 8012, 8050, 8260, 8290, 8343–8344, 8350, 8330–8332, 8335, 8020–8021, 8030–8032)^(26)^. Controls were randomly selected subjects who did physical examination, and they were recruited while they did not have TC in the same period and in the same hospitals, using health screening examinations including thyroid ultrasonography. The inclusion criteria were as follows: age ≥ 18 years, informed about the aim of the study and all participants had been living in Shanghai for at least 6 months at the time of cancer diagnosis. The patients and control subjects were matched in a 1:1 ratio by sex and age (± 2 years). Both cases and controls would be excluded from the study if they had an implausible daily energy intake (< 2,092 kJ/d (500 kcal/d), > 16,736 kJ/d (4000 kcal/d)). Additionally, those participants were excluded from the study if they had history of TC and combination of other cancers, serious mental illness, history of autoimmune disease, pregnant and lactating women or endocrine system diseases (breast disease, ovarian cancer). Four hundred and fourteen pairs of cases (88 % of those eligible) and controls (88 % of those eligible) were included for the final analysis.

This study was conducted according to the guidelines laid down in the Declaration of Helsinki and all procedures involving human subjects were approved by the Institutional Review Board of the Shanghai Municipal Center for Disease Control and Prevention (No. 2015006), and written informed consent was obtained from all participants.

### Data collection

Study subjects were interviewed in person by well-trained interviewers using a standardised, structured questionnaire that covered detailed information on socio-demographic characteristics (e.g. age, education, occupation, household income and marital status), diet, cigarette smoking habits, alcoholic beverages and family history of thyroid diseases. For women, reproductive history including information on menstrual status, age at menarche and menstrual cycle was also obtained. Only exposures or events which occurred before the year of diagnosis for the cases or the year of reference for the controls were taken into account.

Height and weight were measured at the time of recruitment without shoes and with light clothing for three times. All measurements were completed by a trained dietitian. BMI was calculated by dividing weight in kg by the square of height in m (kg/m^2^). Smoking status was determined by smoking behaviours in the previous 30 d before investigation. They were categorised as: (1) current smokers: someone who smoked at least one cigarette every day on average in the past 30 d; (2) ever smokers: someone who ever smoked; (3) passive smokers: participants who did not smoke but were exposed to passive smoking in the home or work place ≥15 min every day and ≥1 d every week; and (4) non-smokers: someone who never smoked. Alcohol intake was computed on the basis of the consumption of beer, rice wine, other wines and hard liquor.

### Dietary assessment

Dietary data were collected using a validated and reliable 121-item FFQ over the 12 months preceding recruitment^(27)^. In terms of FFQ’s reproducibility and validity, the correlation coefficients for food groups ranged from 0·25 to 1·00^(27)^. These food items included: cereals (including noodles, maize potatoes and rice), soyabean, vegetables, fruit, fungus and algae, milk and dairy products, meat and poultry, fish and seafood, eggs, preserved food, alcohol, beverages, snacks, soups, oil and condiments. For each food item, each participant was first asked to report the usual frequency of intake (per day, week, month, year or never) and portion size. All frequencies reported were converted to a daily consumption value. Meanwhile, oil, salt and sugar data were calculated based on the amount of cooking oil, salt, sugar and the food portions reported by the participants who consumed the household condiments together at each meal. The average daily intake of nutrients and total energy was estimated according to the Chinese Food Composition Table, 2009^(28)^.

### Chinese Health Dietary Index score

CHDI, modiﬁed by He *et al*. from the Chinese Diet Balance Index^(25,29)^, was adopted in our study to evaluate the diet quality of participants, which contains thirteen components. These included nine food groups (refined grains, whole grains, dry bean and tuber, total vegetables, dark green and orange vegetables, fruit, dairy, soyabean, meat and egg, fish, shellfish and mollusk), food variety, energy percentage of SFA, empty calories and Na intake, with a score range of 0–5 or 0–10 for each component. The variable, food types reflect the degree of food diversity, and nine food intake indicators are required to evaluate food intake. While energy percentage of SFA reflect the proper selection of high quality protein-source food, empty calories are an indicator for less oil, sugar control and limited alcohol. Refined grains are limitation component, in which a higher intake indicates a lower score. The scoring criteria of the CHDI items used for the study participants are detailed in Table 1.

**Table 1.** Operationalisation of Chinese Health Dietary Index (CHDI) recommendations in a diet-quality score (0–100)

CHDI component	Scoring criteria		Maximum point
	Minimum score	Maximum score	
Food variety	≤ 5 kind	≥ 12 kinds	10·0
Refined grains	0	≥ 100 g/1000 kcal	5·0
Whole grains, dry bean and tuber	0	≥ 40 g/1000 kcal	5·0
Total vegetables	0	≥ 180 g/1000 kcal	5·0
Dark green and orange vegetables	0	≥ 90 g/1000 kcal	5·0
Fruit	0	≥ 110 g/1000 kcal	10·0
Dairy	0	≥ 100 g/1000 kcal	10·0
Soyabean	0	≥ 10 g/1000 kcal	10·0
Meat and egg	0	≥ 50 g/1000 kcal	5·0
Fish, shellfish and mollusk	0	≥ 30 g/1000 kcal	5·0
Energy percentage of SFA	≥ 15 %	< 10 %	10·0
Na	≥ 4 g/1000 kcal	≤ 1 g/1000 kcal	10·0
Empty calories	≥ 40 %	≤ 20 %	10·0
Total points	–	–	100·0

The overall CHDI score is the sum of the thirteen dietary component scores, ranging from 0 (minimum score) to 100 (maximum score). A higher CHDI score indicated greater compliance with the Dietary Guidelines for Chinese, with better variety, adequacy, moderation and overall balance, and therefore a higher diet quality. Three levels of the CHDI score were created *a priori*: low (0–60 points), average (60–80 points) and high (80–points).

### Statistical analysis

Baseline characteristics between the patients and the control subjects were compared using paired *t* tests or Wilcoxon matched-pairs signed ranks test for continuous variables and McNemar’s test for categorical variables. OR and 95 % CI for TC were estimated by univariate and multiple conditional logistic regression models for diet-quality score using the lowest tertile (≤60 points) as the reference group. Models were adjusted for education, income, BMI, smoking, alcohol drinking, history of benign thyroid conditions, family history of thyroid conditions, history of CT examination and energy. Additional adjustment for menopause, menstrual regularity, menarche, history of pregnancy and history of gynaecological diseases. Restricted cubic spline model was used to reveal the potential nonlinear association between CHDI score and TC risk. Based on Akaike information criteria, four knots (at 25th, 50th, 75th and 90th percentiles of CHDI scores) were suggested to fit the models better^(30)^. In the present study, the average CHDI score (60 points) was used as the reference value. *P*_nonlinearity_ was calculated by using a Wald test.

Stratified analyses were performed according to sex (men and women) and age (≤50 and > 50). For analyses with women only, age at menopause and oral contraceptive and oestrogen use were further adjusted for. Statistical significance was inferred for two-tailed *P* values of less than 0·05. All statistical analyses were performed with SPSS software (version 24.0, SPSS) and Stata software (version 15.0, Stata Corp.).

## Results

### Characteristics of cases and controls

Table 2 shows the general characteristics of the cases and controls. Both cases and controls had similar distributions of age and sex. Participants consisted of over two times more women (73·2 %) than men. For both men and women, cases were more likely to have a lower proportion of occupation with a non-manual work, education level but higher proportions of overweight or obese and history of benign thyroid conditions compared with controls (Table 2). In addition, women cases were younger at menopause and tended to use of oral contraceptive and oestrogen. No signiﬁcant differences were observed for smoking status and marital status.

**Table 2.** Distribution of demographic characteristics across cases and controls (Numbers and percentages)

Characteristics	All						Male				*χ* ^2^	*P*	Female					
	Case (*n* 414)		Control (*n* 414)		*χ* ^2^	*P*	Case (*n* 111)		Control (*n* 111)				Case (*n* 303)					
	*n*	%	*n*	%	*n*	%	*n*	%	*n*	%			*n*	%	Control (*n* 303)		χ^2^	*P*
Level of education					25·155	<0·001					1·079	0·583			27·378			<0·001
Junior high school or below	97	23·4	56	13·5			20	18·0	15	13·5			77	25·4	41	13·5		
Senior secondary technical school	161	38·9	135	32·6			43	38·7	42	37·8			118	38·9	93	30·7		
College or above	156	37·7	223	53·9			48	43·2	54	48·6			108	35·6	169	55·8		
Marital status					1·489	0·475					0·474	0·789			1·041			0·594
Never married	34	8·2	44	10·6			9	8·1	12	10·8			25	8·3	32	10·6		
Married/Cohabitation	362	87·4	354	85·5			100	90·1	97	87·4			262	86·5	257	84·8		
Divorced/Widowed /Separate	18	4·3	16	3·9			2	1·8	2	1·8			16	5·3	14	4·6		
Occupation					28·014	<0·001					3·567	0·059			25·435			<0·001
Non-manual workers	156	37·7	232	56			43	38·7	57	51·4			113	37·3	175	57·8		
Manual workers	258	62·3	182	44			68	61·3	54	48·6			190	62·7	128	42·2		
Average monthly household income (¥)					2·792	0·248					4·584	0·101			0·995			0·608
<3000	77	18·6	66	15·9			19	17·1	17	15·3			58	19·1	49	16·2		
3000–4999	137	33·1	159	38·4			31	27·9	46	41·4			106	35·0	113	37·3		
≥5000	200	48·3	189	45·7			61	55·0	48	43·2			139	45·9	141	46·5		
BMI (kg/m^2^)					20·089	<0·001					9·491	0·023			17·881			<0·001
<18·5	19	4·6	26	6·3			3	2·7	0	0·0			16	5·3	26	8·6		
18·5–23·99	232	56	285	68·8			41	36·9	61	55·0			191	63·0	224	73·9		
≥24	163	39·4	103	24·9			67	60·4	50	45·0			96	31·7	53	17·5		
History of CT examination (times)					7·142	0·028					0·948	0·622			8·636			0·013
No	159	38·4	195	47·1			44	39·6	50	45·0			115	38·0	145	47·9		
1–3	204	49·3	182	44			49	44·1	42	37·8			155	51·2	140	46·2		
≥4	51	12·3	37	8·9			18	16·2	19	17·1			33	10·9	18	5·9		
Smoking status					12·923	0·005					8·471	0·037			5·079			0·166
Non-smoker	177	42·8	205	49·5			24	21·6	27	24·3			153	50·5	178	58·7		
Passive smoker	157	37·9	136	32·9			19	17·1	20	18·0			138	45·5	116	38·3		
Current smoker	36	8·7	16	3·9			27	24·3	11	9·9			9	3·0	5	1·7		
Former smoker	44	10·6	57	13·8			41	36·9	53	47·7			3	1·0	4	1·3		
History of benign thyroid conditions					111·131	<0·001					22·676	<0·001			89·971			<0·001
No	240	58·0	373	90·1			78	70·3	105	94·6			162	53·5	268	88·4		
Yes	174	42·0	41	9·9			33	29·7	6	5·4			141	46·5	35	11·6		
Age at menarche															7·489			0·029
≤15 years	/		/				/		/				238	78·5	255	84·2		
>15 years	/		/				/		/				65	21·5	48	15·8		
Ever used oral contraceptives or hormone contraceptive															8·135			0·039
No	/		/				/		/				251	82·8	255	84·2		
Yes	/		/				/		/				52	17·2	48	15·8		

### Thyroid cancer risk associated with Chinese Health Dietary Index components

The median score for each component and the total of the CHDI score are presented in Table 3. The median (range) total scores for cases and controls were 67·5 (58·6–76·7) and 72·8(64·5–80·5), respectively, and the difference between case and control group was signiﬁcant (*χ* ^2^ = 30·794; *P* = 0·003). In comparison with the controls, cases had significantly lower median CHDI scores for fruit (6·8 *v*. 9·3; *P* = 0·004), diary (3·6 *v*. 5·5; *P* < 0·001), soyabean (4·4 *v*. 5·4; *P* = 0·015), whole grains, dry bean and tuber (1·1 *v*. 1·4; *P* < 0·001), total vegetables (3·1 *v*. 4·4; *P* < 0·001), dark green and orange vegetables (3·7 *v*. 5·0; *P* < 0·001), fish, shellfish and mollusk (3·2 *v*. 4·0; *P* < 0·001), whereas the median CHDI score for refined grains among cases was higher than controls (5·0 *v*. 4·9; *P* < 0·001).

**Table 3.** Distribution of the Chinese Health Dietary Index (CHDI) components among cases and controls (Mean values and percentiles)

Items	Points M	P_25_, P_75_	CHDI points (%)			c^2^/Z	*P*	items	Points M	P_25_, P_75_	CHDI points (%)			c^2^/Z	*P*
			0, 5	5, 10	10						0, 2·5	2·5, 5	5		
Food variety						-0·618	0·567	Refined grains						-4·458	<0·001
Case	10·0	8·9, 10·0	8·7	21·0	70·3			Case	5·0	4·5, 5·0	7·0	26·3	66·7		
Control	10·0	8·3, 10·0	8·0	25·4	66·7			Control	4·9	3·5, 5·0	9·4	40·8	49·8		
Fruit						-5·058	<0·001	Whole grains, dry bean and tuber						-3·543	<0·001
Case	6·8	3·2, 10·0	39·6	27·5	32·9			Case	1·1	0·3, 2·0	81·6	11·8	6·5		
Control	9·3	4·8, 10·0	27·1	27·1	47·1			Control	1·4	0·6, 2·6	72·9	19·1	8·0		
Diary						-4·384	<0·001	Total vegetables						-5·942	<0·001
Case	3·6	0·7, 7·6	59·1	23·2	17·6			Case	3·1	1·6, 5·0	40·5	25·8	33·6		
Control	5·5	1·5, 10·0	45·6	27·1	27·3			Control	4·4	2·7, 5·0	19·8	38·2	42·0		
Soyabean						-2·865	0·004	Dark green and orange vegetables						-5·693	<0·001
Case	4·4	1·7,7·7	53·9	30·7	15·5			Case	3·7	1·7, 5·0	36·9	24·2	38·9		
Control	5·4	2·5,9·5	46·6	30·2	23·4			Control	5·0	2·8, 5·0	19·3	30·0	50·7		
Energy percentage of SFA						-1·830	0·067	Meat and egg						-4·754	<0·001
Case	7·1	1·2, 10·0	38·1	25·4	36·5			Case	5·0	4·0, 5·0	11·9	22·5	65·7		
Control	5·9	0·1, 10·0	45·2	24·6	30·2			Control	5·0	5·0, 5·0	4·1	19·1	76·8		
Na intake						-0·323	0·747	Fish, shellfish and mollusk						-3·237	0·001
Case	8·3	6·6, 9·9	16·0	59·9	24·2			Case	3·2	1·4, 5·0	40·8	26·3	32·9		
Control	8·4	6·5, 9·6	12·6	69·1	18·4			Control	4·0	2·0, 5·0	31·2	28·5	40·3		
Empty calories						-0·653	0·514	Total			0, 60	60, 80	≥ 80	30·794	0·003
Case	10·0	10·0, 10·0	/	10·9	89·1			Case	67·5	58·6, 76·7	29·5	53·6	16·9		
Control	10·0	10·0, 10·0	/	11·1	88·9			Control	72·8	64·5, 80·5	14·3	58·5	27·3		

As shown in Table 3, for each component, all participants were then divided into tertiles according to the ranking of their points: low (0–2·5/5 points), medium (2·5/5–5/10 points) and high (5/10 points, the recommendation or restriction). The proportions of refined grains in the case group that achieved full scores (limited intake) were 66·7 %, while those in the control group were 49·8 % (Table 3).

### Thyroid cancer risk correlated with overall dietary quality

**Fig. 1.**
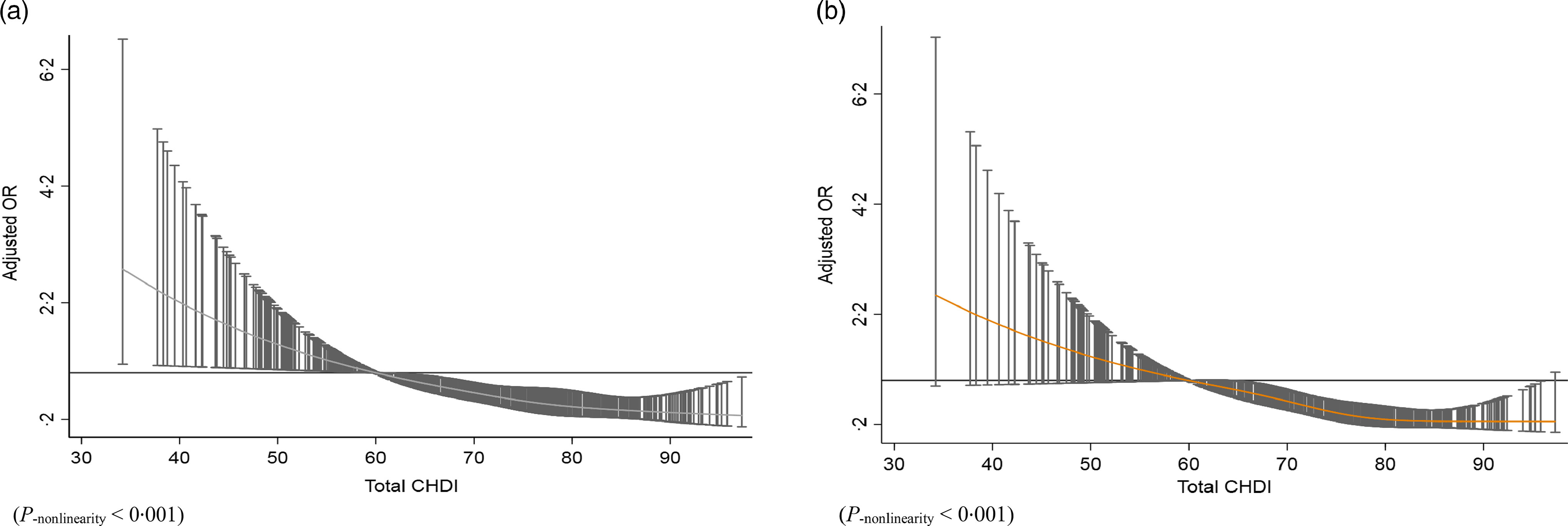
(a) Dose–response association (OR) between thyroid cancer and CHDI scores. (b) Dose–response association (OR) between thyroid cancer and CHDI scores in patients aged younger than 50 years old. CHDI, Chinese Health Dietary Index.

In the average (60–80 points) and high (≥80 points) levels of the CHDI score, the risk of TC was lower by 60 % (OR_adj_ = 0·40, 95 % CI0·26, 0·63) and 78 % (OR_adj_ = 0·22, 95 % CI0·12, 0·38), respectively, when compared with the low level (< 60 points) as a reference (Table 4). After excluding pairs who had a history of benign thyroid conditions, the average and high levels of the CHDI score were inversely related to the risk of TC (OR = 0·33, 95 % CI0·19, 0·57 for average level; OR = 0·22, 95 % CI 0·11, 0·45 for high level), compared with the low level (Table 4). Restricted cubic spline analysis (Fig. 1a) showed a significant L-shaped relationship between CHDI and TC risk (*P*_nonlinearity_ < 0·05), of which with increasing CHDI score, the risk of TC decreased.

**Table 4.** OR for thyroid cancer in relation to Chinese Health Dietary Index (CHDI) scores in overall population, by sex and age strata (Odds ratios and 95 % confidence intervals)

Scores of CHDI	*n* case/control	OR_crude_	95 %CI	*P*	OR_adj_	95 %CI^*^	*P*
Overall							
<60	122/59	1·00		<0·001	1·00		<0·001
60–80	222/242	0·47	0·33, 0·67	<0·001	0·40	0·26, 0·63	<0·001
≥80	70/113	0·31	0·20, 0·48	<0·001	0·22	0·12, 0·38	<0·001
Excluding pairs who had history of benign thyroid conditions							
<60	69/29	1·00		<0·001	1·00		<0·001
60–80	110/126	0·41	0·25, 0·66	<0·001	0·33	0·19, 0·57	<0·001
≥80	34/58	0·27	0·14, 0·49	<0·001	0·22	0·11, 0·45	<0·001
Female^†^							
<60	89/43	1·00		0·001	1·00		0·001
60–80	162/177	0·47	0·32, 0·71	0·002	0·41	0·24, 0·72	0·002
≥80	52/83	0·31	0·19, 0·52	<0·001	0·25	0·12, 0·51	<0·001
Male							
<60	33/16	1·00		0·020	1·00		0·002
60–80	60/65	0·46	0·23, 0·93	0·030	0·35	0·15, 0·79	0·011
≥80	18/30	0·31	0·13, 0·71	0·006	0·15	0·05, 0·44	0·001
Age ≤50 years							
<60	99/39	1·00		<0·001	1·00		<0·001
60–80	183/191	0·40	0·26, 0·61	<0·001	0·31	0·18, 0·54	<0·001
≥80	51/103	0·19	0·11, 0·33	<0·001	0·15	0·07, 0·30	<0·001
Age >50 years							
<60	23/20	1·00		0·306	1·00		0·455
60–80	39/51	0·67	0·35, 1·27	0·215	0·53	0·19, 1·54	0·246
≥80	19/10	1·09	0·47, 2·54	0·845	0·88	0·20, 3·80	0·861

* Multiple logistic regression models adjusted for education, income, BMI, smoking, alcohol drinking, history of benign thyroid conditions, family history of thyroid conditions, history of CT examination and energy.

† Multiple logistic regression models additionally adjusted for menopause, menstrual regularity, menarche, history of pregnancy, history of gynaecological diseases, and oral contraceptive and oestrogen.

CHDI was significantly associated with a lower risk of TC in both men and women. OR (average and high *v*. low) ranged from 0·15 to 0·34 for men and from 0·23 to 0·39 for women (Table 4). We observed inverse relationship between high CHDI and TC risk among women aged younger than 50 years, whereas no significant association was found between high CHDI and TC risk in women over 50 years old. A L–shaped curve with effect for CHDI and TC risk was found (*P*_nonlinearity_ < 0·05, Fig. 1b). There were no statistically significant interactions between age and CHDI score on the risk of TC (*P*_interaction_ > 0·05).

## Discussion

This 1:1 matched case–control study showed the higher scores of CHDI were associated with a reduced risk of TC. Furthermore, a protective effect of this overall diet quality was found among participants younger than 50 years, indicating that the importance of diet quality as a potentially modifiable tool to improve public dietary recommendations among men and women to prevent TC.

As reflecting the synergistic effects of diverse food and numerous nutrients characterised, a high CHDI score is more closely to dietary recommendations and core items of Chinese Dietary Guidelines, indicating greater consumption of fruits and vegetables, whole grain, dairy products, protein and unsaturated fatty acids, as well as a lower consumption of refined grain, Na, added sugars and saturated fats^(31)^. A recent survey about the dietary intake quality of high school students in Shanghai showed that the dietary quality was inappropriate and the CHDI score in urban area was significantly higher than those in suburban and countryside, which might be due to the higher socio-economic status and the better living standards among people in urban areas when compared with those in suburban and countryside^(32,33)^. Another large study to evaluate the dietary quality of Chinese adults reported that the CHDI score among hypertensive population or malnourished population (BMI < 18·5) was significantly lower than those among healthy population or non-hypertensive population^(25)^, suggesting that CHDI could be considered as a potential tool to evaluate the healthfulness of individual diets. The present study showed that high adherence to CHDI was associated with lower TC risk, when compared with the lowest CHDI score, which is consistent with several reports in which high-quality dietary scores showed an inverse relationship to the risk of other cancers^(17–20)^.

Iodine is supposed to be related to TC, and fish and shellfish are an important source of iodine^(34)^. A recent case–control study from Hangzhou, China found an inverse relationship between seafood and cereal pattern and the risk of TC (OR = 0·286, 95 % CI 0·146, 0·561)^(21)^. Also, in a meta-analysis of nineteen case–control studies, Liu *et al.* demonstrated a reduced TC risk associated with ﬁsh and shellﬁsh intake in iodine deﬁciency areas^(11)^, in which the possibility of the biological mechanism was explained by the potential function of iodine-rich intake in protecting against TC^(11,35)^. Shanghai is an environmentally iodine-deficient area, foods rich in iodine should be appropriately added to the diet for reducing the risk of TC. A high consumption of fruit and vegetables is considered one of pillars of a healthy diet. The anti-cancer effects of fruit and vegetables on TC via antioxidant mechanisms have been confirmed by several previous studies^(9,13)^. Fruit and vegetables might also reduce risk of TC via serving as substrates for the formation of anti-neoplastic agents^(36)^, by inducing goitrogens due to thiocyanates production^(37)^, by inhibiting nitrosamine formation, or by altering hormone metabolism^(13)^, and by interacting with other dietary components (iodine intake)^(24)^. Furthermore, soyabean represents an important source of folate, phytochemicals, sterols, glutathione, tocopherols and phenolic compounds, in which these presumed antioxidant properties and anti-cancer properties were correlated with lower risk of cancer of thyroid initiation and progression^(38,39)^.

The consumption of refined cereal and higher sugar foods has been associated with the risk of TC because of their high glycaemic index^(24,41)^. Similar to our results, in a case–control study from French Polynesia, a ‘Western dietary pattern’ (including bread, rice and pasta) was positively related to elevated risk of differentiated TC^(42)^. Given that refined cereals and sugars had a higher rate of digestion than do whole-grain cereals and other components of diet, this might lead to glycaemic overload and compensatory increases in plasma insulin concentration and insulin-like growth factor I, which was an important mitogenic stimulant of tumour cell (TSH thyroid cell) growth *in vitro* ^(43,44)^. Randi *et al*. found that elevated dietary glycaemic index and glycaemic overload could indicate a diet rich in refined carbohydrates (such as rice and ﬁne wheat ﬂour) that have been correlated with TC risk^(44)^. Therefore, in line with our results, dietary guidelines from most countries have either recommended decreasing the total amount of refined cereal or increasing whole grains^(33,45)^.

We further observed a favourable association among participants younger than 50 years, which may be related to the diet-related health education^(46,47)^. A cross-sectional epidemiological study showed that the food construction in 60·0 % of Shanghai elders was unbalanced, including that consuming not enough dairy, fresh fruit and fish^(48)^. Participants younger 50 years were more likely to have high educational levels, and they have more opportunities to earn more money, be likely to have higher rates of literacy and healthier dietary habits^(49)^. On the other hand, the relatively small sample size of participants (case–control pairs, ≤ 50 years old *v*. > 50 years old: 333 *v*. 81) in our study might contribute to the age differences. Future studies in large clinical trials with sufficient statistical power are needed to confirm our findings.

Our study has several strengths and limitations. To our knowledge, this was the first investigation of an association between diet-quality score derived from Chinese Dietary Guidelines and the risk of TC using a well-constructed FFQ with satisfactory reproducibility and validity^(50)^. A fully adjusted model for the diet–cancer relationship was also made in the multivariate analysis for a broad range of potential confounders. Sensitivity analysis excluding pairs who had history of benign thyroid conditions did not materially change the result. However, recall bias is susceptible to the case–control design. To minimise this possibility, only newly cases were included in our study, and both TC patients and healthy controls were confined to those without a substantial change in dietary habits over the previous 12 months. A 1:1 matched study was also designed to minimise the influence of age and sex.

### Conclusions

In conclusion, the present study provided interesting insight into the beneficial effects of high and average dietary scores on TC risk in Eastern of China. This dietary score might be a useful and convenient tool for the reduction of TC. Prospective studies are warranted to validate our findings.
